# A Review of Machine Learning and Deep Learning Methods for Person Detection, Tracking and Identification, and Face Recognition with Applications

**DOI:** 10.3390/s25051410

**Published:** 2025-02-26

**Authors:** Beibut Amirgaliyev, Miras Mussabek, Tomiris Rakhimzhanova, Ainur Zhumadillayeva

**Affiliations:** Department of Computer Engineering, Astana IT University, Astana 010000, Kazakhstan; beibut.amirgaliyev@astanait.edu.kz (B.A.); 242677@astanait.edu.kz (M.M.); tomiris.khalimova@nu.edu.kz (T.R.)

**Keywords:** computer vision, video analysis, deep learning, person detection, person tracking, person identification, face recognition

## Abstract

This paper provides a comprehensive analysis of recent developments in face recognition, tracking, identification, and person detection technologies, highlighting the benefits and drawbacks of the available techniques. To assess the state-of-art in these domains, we reviewed more than one hundred eminent journal articles focusing on current trends and research gaps in machine learning and deep learning methods. A systematic review using the PRISMA method helped us to generalize the search for the most relevant articles in this area. Based on our screening and evaluation procedures, we found and examined 142 relevant papers, evaluating their reporting compliance, sufficiency, and methodological quality. Our findings highlight essential methods of person detection, tracking and identification, and face recognition tasks, emphasizing current trends and illustrating a clear transition from classical to deep learning methods with existing datasets, divided by task and including statistics for each of them. As a result of this comprehensive review, we agree that the results demonstrate notable improvements. Still, there remain several key challenges like refining model robustness under varying environmental conditions, including diverse lighting and occlusion; adaptation to different camera angles; and ethical and legal issues related to privacy rights.

## 1. Introduction

In recent years, the rapid development of artificial intelligence (AI) has facilitated its application across numerous industries. One such sector is real-time people monitoring, which encompasses person detection, identification, and tracking systems—where ensuring safety, efficiency, and the overall well-being of individuals are of crucial importance. Real-time people monitoring systems have become a crucial task for governments and companies. Such systems can serve as surveillance systems or analytical tools that help to understand people’s behaviors and intentions. However, they require a significant number of cameras to cover areas with crowds of people and monitor video streams in real-time without interruption. Given the scale of this task, manual monitoring is impractical. As integral components of AI, machine learning (ML) and deep learning (DL) have emerged as crucial solutions that significantly enhance these systems.

These systems leverage a combination of advanced techniques, including computer vision (CV) and the Internet of Things (IoT), to observe and analyze people’s behaviors, movements, and interactions in real-time. Particularly, CV technologies in people monitoring have notably enhanced security and safety over time. Moreover, more advanced systems are capable of counting people [1], recognizing individuals [2], and alerting security or emergency personnel to potential threats [3,4]. These examples illustrate the significant impact of ML and DL on people monitoring, highlighting their effectiveness and the need for ongoing integration.

Despite the promising potential of CV technologies, their rapid development presents several challenges and limitations. These include challenges related to accuracy, handling different camera poses and positions, and delivering real-time performance. Therefore, it is essential to critically examine the current trends and technological advancements in this field while also identifying their limitations to highlight areas requiring further research. Also, implementing DL and ML systems is an interdisciplinary approach, covering not only technological aspects such as image processing, computational efficiency, and data analytics but also social aspects. These include the ethical implications of automation, privacy concerns, and the social implications of adopting such technologies in various sectors. One primary concern is privacy, particularly regarding access to personal data, tracking movement patterns, and contacts. For instance, in [5], the authors propose using blurred images to preserve privacy in human detection. Additionally, several studies highlight issues such as privacy risks associated with data collection, dataset bias, and the potential for misuse of the technology. These concerns not only pose limitations but also drive the development of new algorithms that incorporate ethical considerations [6,7].

This literature review aims to comprehensively analyze the current state of machine learning and deep learning methods for person detection, tracking and identification, and face recognition. Rather than introducing new experimental research, our review synthesizes existing studies by examining different technologies that have been used in these systems. Also, it highlights the key applications of various modes and discusses the associated challenges and limitations. By summarizing existing research, this review aims to evaluate progress in this area and suggest new directions for improving the safety and effectiveness of occupant monitoring systems.

The contributions of this review are as follows:

- We present a comprehensive review of ML and DL methods for person detection, tracking, identification, and recognition, describing the current technologies and future challenges in the field.
- We reviewed and summarized nearly 35 scientific publications on CV detection systems, focusing on key methodologies from 2014 to 2024. These publications are categorized according to different computer vision approaches, such as people detection, tracking and identification, and face recognition.
- We analyzed and compared prominent DL architectures and their applications, specifically focusing on their implementation and performance across metrics such as real-time accuracy, reliability across varying conditions, and effectiveness in recognizing complex human behaviors.
- We discuss potential future directions in the field and highlight trends and areas where further research could have a significant impact.

## 2. Methodology

In this review, we followed the PRISMA (Preferred Reporting Items for Systematic Reviews and Meta-Analyses) framework to conduct a comprehensive literature search, apply study selection criteria, and extract specific data. PRISMA was initially created for researchers who perform systematic reviews to aid in transparent reporting, answering questions about why the review was performed, what precisely the authors did, and what they concluded [8]. It is the updated version of the version published in 2009 [9]. The PRISMA 2020 guidelines consist of seven checklist items and a flow diagram showing the number of records identified, included, and excluded during each selection stage. Following the PRISMA item checklist, our selection process started with identifying databases and a search strategy. We searched across the IEEExplore, ScienceDirect, Google Scholar, and arXiv databases. Using keywords such as “Computer Vision”, “Deep Learning”, “Face Recognition”, “Person Identification”, and “Object Detection Models”, we aimed to capture relevant studies published within the last ten years. Although we tried to include only recent papers, we could not omit old articles with original information. The search was further refined by filtering results to include only journal articles and conference papers, ensuring a focus on peer-reviewed and academically rigorous sources. Each team member worked independently, except on extensive relevant articles, where we worked cooperatively.

**Figure 1:**
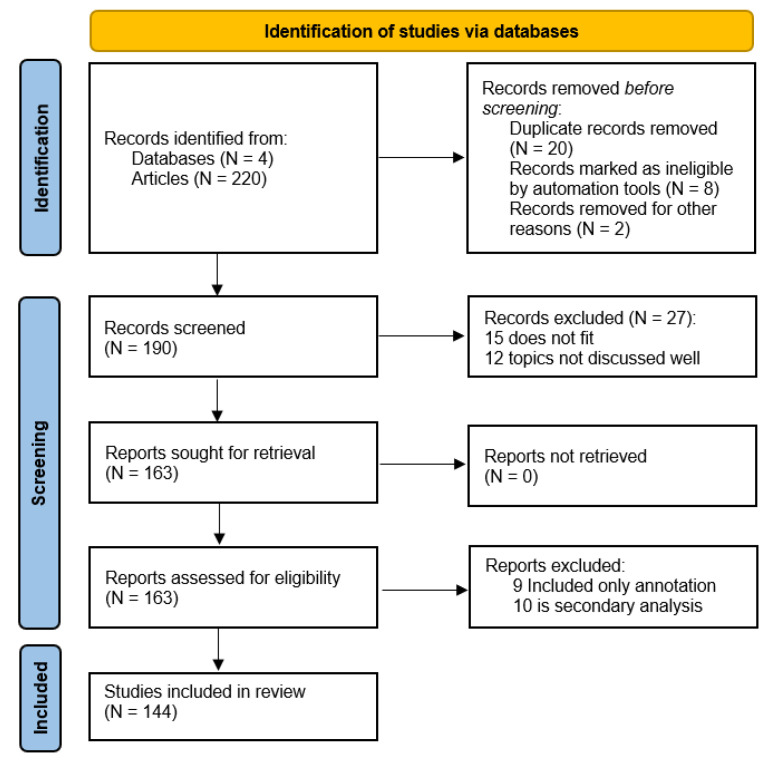
Table of used PRISMA model in this review.

We created a pool of 220 peer-reviewed papers. We aimed to include articles published within the last 10 years to include only relevant and up-to-date information. However, we also included some earlier publications since they were considered original and provided foundational insights not covered in more recent literature. After an initial screening based on titles and abstracts, 163 articles were retained for further evaluation. We applied strict inclusion criteria, focusing on studies that addressed specific technological approaches in methods for person detection, tracking and identification, and face recognition, or those that presented experiments involving deep learning models. Only papers with robust methodologies and clear relevance to the study objectives were included. Exclusion criteria were used to remove articles that were either too general, relied on outdated technology, or presented biased or irrelevant data. Ultimately, 144 high-quality studies were selected for inclusion in our review (see Figure 1).

## 3. Person Detection, Tracking and Identification, Face Recognition

This study focused primarily on complex ML and DL methods with their applications in person detection, tracking and identification, and face recognition tasks. Although concepts like image preprocessing, feature extraction, and classification can be used to solve these problems as standalone solutions [10], some of them are already part of more complex and modern models. For example, we can point out the YOLO (You Only Look Once) [11] object detection model with integrated inner parts. In the third version of YOLO, developers composed a feature extractor part with 53 convolutional layers, which became a multi-scale feature extraction architecture and one of the essential steps in object detection.

### 3.1. Person Detection

One of the most critical tasks in computer vision is person detection, which involves locating and identifying individuals in images or video streams. Person detection is generally a subset of the object detection problem with the limitation of locating human figures. Human-like objects are highlighted and set apart from the background by surrounding them with a rectangular frame. All models in object detection are divided into two categories based on their detection type: single-stage or two-stage methods. Two-stage object detection methods divide the object classification task from the object location task and, prior to classifying the region, generate the region proposal [12]. They first utilized Deep Convolutional Neural Networks (DCNNs), which showed high detection accuracy but with slow detection speed. With the advancement of technology and new larger datasets, single-stage DCNNs were introduced. Their main advantage was real-time processing speed, but they are less accurate, especially for small objects in low-resolution images [5]. In addition to detection speed and small objects, other problems like dense occlusion can occur, where the model often leads to missed and false detections, as in pedestrian detection, particularly when objects of the same or different categories obscure one another [12]. Also, the hierarchical structure of CNNs makes detecting objects with multiple scales difficult. This is because classification and bounding-box regression on the final layer of feature maps result in a significant loss of small object feature representation. Class imbalance in one-stage object detection has a lower accuracy compared to two-stage methods. For example, to address the challenge of detecting small people at sea with harsh lighting conditions, the Chinese Academy of Sciences created their benchmark, referred to as TinyPerson [13]. Their dataset contained 72,651 annotated images with people near the sea, then it was replaced with a new version to directly work with the people in images [14]. Furthermore, post-processing methods like Non-Maximum Suppression (NMS) are required to remove duplicates and preserve the most accurate bounding boxes due to the redundancy in bounding boxes, while more recent algorithms like Soft-NMS and IoU-Net improve the localization accuracy of the detections [15].

Another method that provides much more detailed information at the cost of higher computational complexity is detection via segmentation. Unlike object detection, which uses bounding boxes to locate people, segmentation provides pixel-level accuracy, outlining individuals’ exact shapes and contours. Overall segmentation can be categorized into three primary types: instance segmentation, semantic segmentation, and panoptic segmentation [16]. Instance segmentation mainly focuses on creating masks around each object to recognize and distinguish distinct objects within an image. In contrast, semantic segmentation assigns a class label to every pixel in the picture, gathering all pixels of the same class under a uniform label. For example, in person detection, instance segmentation can mask unique color-coded masks for each person to avoid confusion, and semantic segmentation might label all pixels belonging to people with a single color. These two methods are combined in panoptic segmentation, supplying clear object boundaries and pixel-by-pixel labels simultaneously. Segmentation technologies rapidly evolve with deep learning and computational power advancement, making person detection more accurate in complex settings. In recent research, the authors proposed a high-efficiency person segmentation system that significantly improves segmentation accuracy while utilizing a much smaller CNN network [17]. In the other research, the authors proposed a new architecture based on MobileNetV3m, which segments persons in images and videos at 35 frames per second on a Google Pixel 4 [18]. Even with improvements in segmentation for person detection, there are still several vital problems similar to object detection, like occlusion in crowded settings and appearance variability brought on by clothing and lighting. Furthermore, many studies are not reproducible because they frequently report results on non-standard datasets or do not clearly specify their experimental setups.

**Figure 2:**
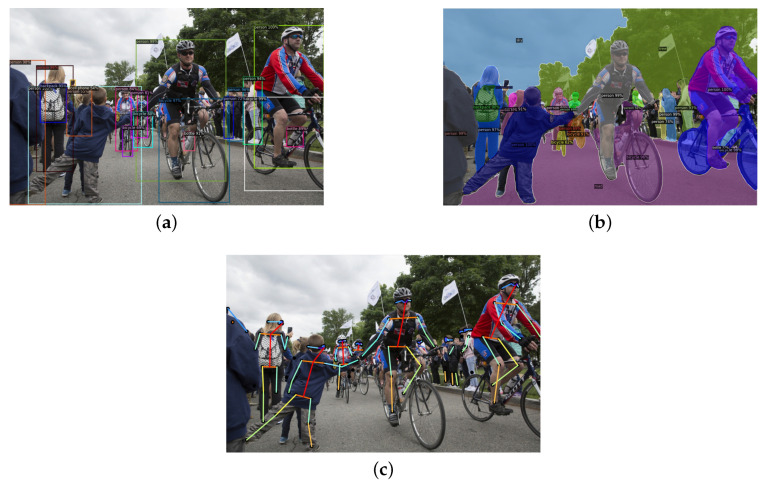
(**a**) Example of object detection, where the model identifies and locates objects in an image using bounding boxes. (**b**) Example of segmentation, where the model assigns pixel-level labels to different regions of the image. (**c**) Example of pose estimation, where the model forecasts the locations and orientations of a person’s major body joints [22].

The last method suitable for person detection tasks is pose estimation. Pose estimation plays a notable role in CV and extends the concepts of person detection by focusing on accurately identifying and localizing the key points of the human body in images or videos. While person detection involves recognizing individuals within a scene, and segmentation aims to delineate their shapes, pose estimation goes a step further by mapping the precise positions of joints and limbs, allowing for detailed understanding of human posture and movement (see Figure 2). Pose estimation is separated into two parts, 2D and 3D pose estimation, where the difference is whether key points are localized in two-dimensional or three-dimensional spaces. Although human pose estimation has advanced significantly, there are issues, particularly when handling complex backgrounds and different person scales. Great importance was attached to architectures like OpenPose, which differentiates between both large and small keypoints [19]. Frameworks such as UniPose+ leverage multi-scale feature representations and enable accurate 2D and 3D pose estimation without increasing computational complexity [20]. Both models impose efficient pose estimation with high accuracy, but OpenPose is a bottom-up approach. The model detects all body points in an image then groups them for each person, making it computationally expensive. Additionally, it struggles with occlusions. On the other hand, UniPose+ employs a top-down approach, detecting the person before predicting human body parts. It can be slower in multi-person scenarios but still achieves state-of-the-art results. Additionally, client–server architectures have been used to create real-time mobile solutions that enable quick and low-computation pose tracking [21].

### 3.2. Person Tracking

Person tracking involves continuously following people across many video frames. The problem extends the object detection model’s usability by identifying bounding boxes around people and associating detections from one frame to another. Early tracking methods relied on traditional methods like background subtraction and optical flow, but they cannot deal well with occlusions or crowded environments. However, experts are now able to build more accurate and robust tracking systems through the use of CNNs.

Object tracking, in general, is divided based on the ability of the model to track, in our case, one or many persons via Single Object Tracking (SOT) and Multiple Object Tracking (MOT). SOT systems mainly create complex appearance and motion models to handle difficult situations like scale changes, out-of-plane rotations, and illumination variations [23]. However, modern analytics or surveillance systems are designed to work in complex scenarios. These scenarios often involve crowded environments, occlusions, and multiple-person interactions, making SOT impractical.

Available systems for use like MOT usually include a detection step, whereby targets within individual video frames are located, and an association step, where identified targets are linked to their trajectories [24]. Additionally, the real-time features of multiple object tracking systems are proposed and can be used as surveillance systems. An example of a real-time multi-object tracking algorithm proposed in [25] is the combination of high-speed detections from the YOLO framework with deep feature extraction from a convolutional neural network. The vast utility of tracking technologies was in the context of sports analytics, where the new modified algorithm is proposed for multi-target trajectory tracking [26]. Combining the multi-target detection results from the detection link allows for data association and tracking. The best target center point coordinates for this target type are then entered into the Kalman filter to predict the center point at the subsequent time or multi-target trajectory prediction. Studies in people tracking challenge long-term occlusions and distinguishing between similar persons, improving the accuracy of the models every time [27]. Therefore, the robustness of tracking systems in harsh environments and integration of multi-modal data, such as from depth or temperature sensors, remain significant research gaps in the field.

### 3.3. Person Identification

Person identification is another computer vision task that aims to match a person’s identity in a given frame with database information. Unlike person tracking, which focuses on continuously following a person within a scene, person identification involves accurately matching an image to those from an identity database. Person identification extends to a more challenging task, person re-identification (Re-ID), which requires identification across multiple cameras or locations in a video or image sequence. These systems can be categorized into two main settings: closed-world and open-world. The closed-world setting assumes single-modality data with sufficient, correctly annotated training data, enabling models to operate under well-defined constraints. In comparison, the open-world setting involves heterogeneous, multi-modal data sources, such as raw images or videos, often collected in uncontrolled environments. This environment necessitates that the models handle ambiguity, generalize beyond pre-defined classes, and adapt to new scenarios due to the inclusion of previously undiscovered categories, dynamic data distributions, and sparse or noisy annotations [28]. Person re-identification has seen significant success in the closed-world setting with deep learning techniques centered around metric learning, deep feature representation, and ranking optimization. In the beginning, the most commonly used CNN-based models were the classification model and Siamese model, both image-based re-identification methods [29]. However, as performance saturates, research has moved to the more difficult open-world settings, where differences in clothing, surroundings, and hidden identities create more difficulties representative of real-world applications [28]. Consequently, video-based Re-ID improved, where each identity is represented by a video sequence, requiring either a multi-match strategy or a pooling-based approach for aggregating features across frames [29]. One of the most recent studies showed the ability of Re-ID systems to solve problems with people who change clothes and contributes to the cloth-changing person re-identification (CC-ReID) field [30]. They provide a Component Reconstruction Disentanglement (CRD) module that uses the reconstruction of human component regions to separate the features related to clothing from those that are not related. To be more precise, it has a human parser for region extraction and an edge detector to reconstruct the contours of the human body, so it also regularizes the disentanglement process. Another study introduces the Clothing-Change Feature Augmentation (CCFA) model to augment CC Re-ID data in the feature space [31]. It improves the robustness of variations in clothes through a three-step process, including statistical modeling, feature augmentation generation, and ID-correlated training strategy. However, the same challenges are still persistent, including handling extreme variations in appearance due to occlusion, lighting, or re-identifying people in completely different camera networks. Research gaps also exist in developing more robust algorithms to adapt to new identities in real-time and integrate multi-modal data from different camera types.

### 3.4. Face Recognition

Facial recognition has emerged as a critical technology with a wide range of applications, from security and surveillance to personal identification and authentication. The field has seen significant advancements in recent years, driven by the development of powerful machine learning algorithms, the availability of large-scale facial datasets, and the increasing processing power of modern computing systems [32]. However, despite the considerable progress, several challenges and limitations remain, particularly regarding robustness, fairness, and generalization across diverse conditions.

Face recognition involves several stages, from image capture to final face identification: image capture, preprocessing, face detection, face alignment, feature extraction, comparison, and identification. Each stage uses its methods, models, and algorithms. For example, after capturing an image from a camera or a static photo, it undergoes preprocessing to improve its quality and prepare it for recognition. Then, classical methods such as Haar cascades [33] or histogram of oriented gradients [34] or modern CNN-based models such as MTCNN [35] are used to detect and highlight the face in the image. Face alignment can be achieved using methods that use facial landmarks, which allow the face to be correctly positioned relative to the image axis [36]. After face detection and alignment, features that describe the unique characteristics of the face are extracted. Again, feature extraction can be performed using classical methods that analyze the texture of the face and its geometric features such as LBPs (Local Binary Patterns) [37], as well as using deep neural networks such as FaceNet or VGG-Face, which can extract more complex and deeper features, creating a compact vector representation of the face embedding [38].

Accurate facial recognition often depends on precise facial landmark detection and alignment. Methods like Dlib and OpenFace detect key points on the face (e.g., eyes, nose, and mouth) to align facial images, reducing variations caused by pose, lighting, or expression [39]. These techniques enhance recognition accuracy by standardizing the input before feeding it into a neural network.

**Figure 3:**
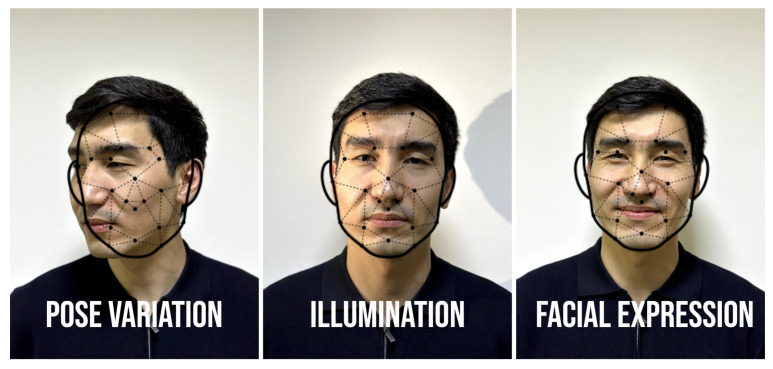
Face recognition challenges due to variations in pose, lighting, and facial expression (image of one of our team members).

One of the primary challenges in face recognition is handling variations in pose, illumination, and facial expression (see Figure 3). While deep learning models have significantly addressed these factors, extreme conditions (e.g., side profiles, low lighting) still pose difficulties [40]. Approaches like 3D face modeling and pose-invariant face recognition are being explored to mitigate these issues. Many face recognition datasets are biased toward certain demographics, particularly regarding race, gender, and age. Studies have shown that face recognition systems perform better on lighter-skinned individuals and males, raising concerns about fairness and potential misuse [41]. Solutions like fair representation learning and debiasing techniques are being developed to address these ethical concerns. Face recognition systems are vulnerable to adversarial attacks, where slight perturbations to an image can mislead a model into making incorrect predictions. Spoofing attacks, such as presenting photos or masks to the system, pose security risks. Adversarial defense mechanisms and liveness detection techniques (e.g., detecting blinking, heartbeat, or texture analysis) are active areas of research aimed at improving the robustness of these systems [42].

Facial appearance changes significantly over time due to aging, which challenges long-term face recognition systems. Although some aging-invariant face recognition models exist, they are far from perfect. Age progression modeling and temporal adaptation methods are being studied to address this issue [43].

Another significant challenge is handling partially obscured faces. In real-world settings, faces may be partially obscured by accessories (e.g., hats, glasses, masks) or objects (e.g., hands or hair), making the recognition task more challenging.

Some facial recognition systems have begun using multimodal data, such as combining facial data with voice or behavioral biometrics, to improve accuracy. Multimodal authentication can improve the robustness of systems. Still, it also introduces new challenges related to the synchronization and processing of different types of data, requiring the development of efficient methods for integrating multimodal data.

## 4. Methods and Materials

### 4.1. Datasets

In deep learning, selecting the appropriate dataset is crucial for training models effectively. Many datasets are available for human detection, tracking and identification, and face recognition. This section will explore the most popular and widely used datasets across these domains, providing a comprehensive look at the resources (see Table 1). The provided datasets are designed to support various real-world applications, including traffic management, surveillance systems, sports analytics, retail, and customer analytics.

#### 4.1.1. Human Detection

The AI City Challenge (AIC) [44] dataset for motorbike helmet violation detection is designed to enhance automated traffic safety enforcement by identifying motorcyclists without helmets. The dataset consists of 100 training videos, each 20 s long at 10 fps and with a resolution of 1920 × 1080. It includes annotated bounding boxes for motorcycles and riders, classifying them based on whether they are wearing a helmet. The benchmark uses mean average precision as the evaluation metric, following the PASCAL VOC 2012 standard, to show reliable performance measurement of the detection models.

PeopleSansPeople [45] is a data generator used to solve issues such as privacy and security in human-centric datasets. The generator creates 3D images with accompanying 2D and 3D annotations of the human localization coordinates in the image. The data also contain standardized pose labels and semantic segmentation information.

The COCO [46] dataset is one of the most popular datasets in computer vision, widely used for object detection tasks and human segmentation tasks. It includes 250,000 human-class images, among many other object categories. Each image contains a corresponding bounding box with the person’s location, key points, and pixel-wise segmentation masks. Due to its scale and complexity, COCO is often used to compare models in people detection, segmentation, and keypoint localization tasks.

The INRIA Person Dataset [65] is specifically designed to aid in developing pedestrian detection models, particularly for applications such as autonomous driving. It contains images of pedestrians and their precise location in the image.

#### 4.1.2. Human Tracking

The created task, the MOT challenge [48], offers researchers a set of videos with complex scenarios for tracking multiple people simultaneously. The dataset consists solely of videos with detection results from benchmark models, offering a valuable resource for assessing tracking algorithms. Another significant dataset for human tracking is the SportsMOT dataset [50]. This dataset represents player movements in football, basketball, and volleyball and consists of 240 video sequences with over 1.6 million bounding boxes and more than 150,000 frames. Because of its distinct features, including fast and variable-speed motion and similar but distinct appearances, SportsMOT presents serious difficulties for both motion-based and appearance-based object association.

The PoseTrack [52] is a vast and extensive dataset containing over 500 videos. These videos are carefully annotated with keypoint coordinates representing points on the human body and detailed tracking labels, making them a rich resource for complex research papers.

#### 4.1.3. Human Segmentation

The COCO dataset remains a primary benchmark for human segmentation, with the highest mask AP score reaching 56.1 [56]. Additionally, the Cityscapes dataset has been pivotal for comparative analysis, where researchers can experiment with urban street scenes. The best-reported mask AP was 38.0 for validation, demonstrating the model’s applicability in city landscape scenarios [55].

SA-1B (Segment Anything 1 Billion) [57] is an extensive dataset comprising real-world high-resolution RGB images totaling over 1 billion in quantity. Every image is annotated with mask-based annotations, making it a good choice for segmentation tasks.

#### 4.1.4. Face Recognition

In the realm of facial recognition, some datasets have been discontinued due to data privacy and security concerns, restricting access to specific previously available resources. Among the currently available datasets is Labeled Faces in the Wild (LFW) [58], which includes over thirteen thousand cropped RGB images. This dataset was created by researchers from the University of Massachusetts in 2007. The dataset was developed to evaluate face verification models, mainly focusing on solving challenges related to varying lighting conditions and poses.

CelebFaces Attributes (CelebA) [60] is a dataset designed for face recognition tasks, which includes images of celebrities. The dataset contains more than ten thousand unique identities without their names and annotations for facial attributes and landmark locations. Similarly, the YouTube Faces database includes 3425 videos of more than 15,000 unique individuals sourced from the YouTube platform. This dataset is presented in h5 format, featuring full-size cropped images in a numpy array format with corresponding annotations for each person’s unique ID.

The large dataset for face recognition is the VGG face dataset [63], which contains more than 2.6 million images with more than 2.6 unique faces. This dataset was collected from the internet, and each annotation includes the image’s URL and face coordinates obtained through detection models, making it an excellent resource for deep learning face recognition applications.

With the rapid development of computer vision technology, researchers are constantly developing new methodologies for human detection, tracking, and segmentation. A critical aspect of evaluating these methods is using standardized metrics, which allow the performance of different models and datasets to be fairly compared and assessed. It is important to note that performance metrics can vary significantly across datasets due to differences in dataset cleanliness and complexity. More complex datasets contribute to developing advanced AI models capable of functioning effectively in diverse and challenging environments.

### 4.2. Classical Computer Vision-Based Methods

Early detection and recognition method development focused on detecting hand-crafted features using fundamental methods. Before CNN development, one of the most well-known method, proposed by Viola and Jones, was the Haar cascade algorithm [66]. In addition to its main usage in face recognition tasks, the Haar cascade was one of the first used as a detection algorithm. It gained popularity for its fast feature evaluation process compared with other detection methods due to the integral image method, which allowed for feature evaluation. Adding an integral image to the cascade classifier makes this method possible for real-time usage. Combined with the AdaBoost algorithm, the consumption of computing resources by the Haar cascade algorithm can be reduced [65]. However, the complexity of new practical datasets became higher, so the Haar cascade could not compete with other methods. Then, the Histogram of Oriented Gradients (HOG) feature descriptor was proposed by Dalal and Triggs [67]. Regarding human detection, HOG maintains fine orientation sampling, which deals with many different human edge directions, and robust local photometric normalization, so lightning conditions will not have a significant effect. The Histogram of Oriented Gradients method has been refined to enhance person detection accuracy. This improvement involves generating more detailed descriptors by integrating additional features, such as color and texture information [68,69]. Additionally, combining HOG with the Support Vector Machine (SVM) algorithm has proven to be a highly efficient approach for human classification tasks, utilizing the extracted descriptors to classify whether or not an image region contains a person [70]. However, it struggles with small objects because coarse spatial sampling cannot capture enough meaningful detail, and it struggles if a person changes pose. On the other hand, the Deformable Part-Based Model (DPM) was introduced to improve the handling of variations in object shape and pose. It is more robust than rigid template-based methods since it represents objects as a collection of deformable parts [71]. The model used a latent SVM for classification and an efficient dynamic programming approach for part-based matching. The main drawback of DPM is that it is computationally expensive due to multiple HOG filters, which are used in different locations of the images (see Table 2).

Optical flow has been widely used for tracking moving objects across a sequence of frames. As described in [79], this technique estimates motion by analyzing the changes in pixel intensities between consecutive frames, providing valuable insights into object dynamics. In applications like passenger monitoring [80,81], optical flow can capture flow patterns and behaviors by detecting movement, making it a crucial tool for understanding traffic or crowd dynamics. Combining SIFT for identifying static key points and optical flow for dynamic tracking enhances system performance in a wide range of object detection tasks [82]. Recent articles include the evaluation of new datasets with methods based on optical flow. One is the Skipped-Detection and Optical-Flow Tracker (SDOF-Tracker), which achieves more robustness by incorporating intermittent detections and selecting strategic tracking points [72]. The authors tested it on the MOT20 dataset. Another method that can enhance human tracking is the Kalman filter, which helps reduce noise, associates multiple objects with tracks, and identifies objects within an image. However, studies suggest that the Kalman filter performs best when combined with other techniques, such as image segmentation [83] or the HOG [84], to improve tracking accuracy and robustness. As this paper [85] suggests, mean shift, which locates local minima of a similarity metric between the target image and the model’s color histograms, proved to be an approach with good object tracking performance. The combination with Continuously Adaptive Mean Shift (CAMshift), which adapts to object size and shape changes, further improves tracking by enabling real-time data updates and providing robustness to occlusions and appearance changes. For instance, CAMshift tracking possibilities were tested on a custom dataset in ref. [74], and in ref. [86], the authors combined the CAMshift algorithm with the YOLO network and a Kalman filter to detect people in a real-time, challenging environment, where the system showed promising results.

It is generally accepted that the foundation paper in person identification and re-identification was [87], which proposed a ranking-based approach to match individuals across different camera views. The problem with a person disappearing temporarily due to occlusions or scene changes proposes a large area for research. Soon, it laid the groundwork for many popular algorithms like Ensemble RankSVM, which enhanced robustness by combining multiple Support Vector Machines (SVMs) to improve pairwise ranking [88]. Unlike traditional classification methods, RankSVM is ideally suited for ReID assignments where exact ordering is essential since it places a more significant priority on rating-related identities. However, a drawback of Ensemble RankSVM is its high computational cost during inference, as multiple SVMs must be evaluated per comparison, and it scales poorly for large datasets or real-time applications. Similarly, Symmetry-Driven Accumulation of Local Features (SDALF) has demonstrated strong performance in person re-identification tasks by leveraging domain-specific features. SDALF utilizes Maximally Stable Color Regions (MSCR), Weighted Histograms of Oriented Gradients (WHOG), and Recurrent High-Structured Patches (RHSP) to capture color, shape, and texture information while exploiting the symmetry of the human body. This approach makes SDALF robust to variations in lighting, pose, and viewpoint, enabling reliable matching of individuals across non-overlapping camera views. Its focus on symmetry and handcrafted features has made it a competitive baseline method for re-ID, particularly in scenarios with limited training data [76]. Another technique for person ReID is Custom Pictorial Structure (CPS) [77], which is the improved version of Pictorial Structure (PS) from ref. [89]. It uses Pictorial Structures (PS) to localize and match body parts in single images. When multiple images of an individual are available, CPS customizes the PS model to learn the person’s appearance, improving part localization and re-identification accuracy through statistical learning of pixel attributes and spatio-temporal reasoning. This approach achieves state-of-the-art results and opens new research directions in re-identification.

The Eigenfaces method was one of the first successful face recognition techniques [90]. It used Principal Component Analysis (PCA) to represent face images as a combination of principal components or eigenfaces. This dimensionality reduction technique allowed for efficient face recognition by capturing the most significant variations in facial appearance. However, while Eigenfaces worked well under controlled conditions, it was sensitive to lighting, pose, and expression changes, making it less effective in real-world scenarios. To overcome the limitations of Eigenfaces, Fisherfaces was introduced using Linear Discriminant Analysis (LDA) instead of PCA [90]. Unlike Eigenfaces, which captures the most significant variance in the dataset, Fisherfaces focuses on maximizing the separation between different classes (faces of different individuals). This improvement made Fisherfaces more robust to illumination changes and facial expression variations, as it better preserved identity-related information. However, it struggled with large pose variations and required well-aligned face images for accurate recognition. While Eigenfaces and Fisherfaces relied on statistical transformations, Gabor wavelets [91] introduced a more biologically inspired approach. These filters are designed to mimic the human visual system’s sensitivity to spatial frequency and orientation, making them particularly effective for capturing fine-grained facial details. By analyzing texture at multiple scales and orientations, Gabor wavelets significantly improved face recognition under different lighting conditions. However, despite their robustness, they came at a cost, high computational complexity, as multiple convolutions were needed to extract features.

### 4.3. Deep Learning-Based Methods

With advancements in computer vision, this field has gained implementation in monitoring systems, especially for person detection, tracking, identification, and face recognition. These deep learning architectures are widely used for tasks like behavior recognition and facial expression analysis. Although these systems have shown promising results, they still need to be improved, particularly in terms of robustness when operating under challenging environmental conditions.

Deep learning is well-suited for handling real-time video streams, where swift and accurate analysis is essential. This demand has led to the standardization of face and object recognition algorithms, with convolutional neural networks being particularly effective. CNNs excel at tasks like object detection [92], classification [93], and face recognition [94] due to their ability to capture spatial hierarchies of features in images, enabling robust and precise analysis in complex, high-traffic environments (see Table 3). One notable example is ref. [95], where the authors propose combining CNNs with a spatio-temporal approach for human counting. This method addresses challenges in the field, such as people identification in high-density environments and short detection times, making it highly suitable for real-time applications. The use of 3D CNNs is discussed in ref. [96], where the authors apply it in combination with transfer learning, utilizing data from two cameras to classify passenger actions such as drinking, calling, and eating. However, the authors note that this approach has limitations in detecting small or subtle movements.

To address this issue, researchers have explored the use of CNNs with other network features such as long short-term memory (LSTM) blocks to capture temporal information. This combination demonstrates precise accuracy and performance in the different implementations, including vandalism detection [97] and human activity recognition [98]. The authors of ref. [99] employ a CNN–LSTM neural network for tasks such as human counting, vehicle localization, and detecting unattended children. Additionally, this combination has proven effective in addressing occlusion challenges in multi-object tracking scenarios [100]. The method of using CNN and LSTM layers together is beneficial for decreasing computational complexity [97]. These papers highlight both the necessity of combining CNNs with other methods to enhance accuracy and the everyday challenges encountered in this field.

**Table 3:** Deep learning-based methods mapped to primary problem domains.

Domain	Method	Key Features	Performance Metrics
Person detection	YOLOv4	Fast, real-time capable	43.0% AP [0.5:0.95] on MS COCO [101]
	Faster R-CNN	Two-stage detector, high accuracy, region proposal network (RPN), slower	27.2% AP [0.5:0.95] on MS COCO [101]
	SSD (Single Shot Detector)	Single-stage, fast, good trade-off between speed and accuracy, multi-scale features	26.8% AP [0.5:0.95] on MS COCO [101]
	Few-Shot Detection Transformer (FS-DETR)	Combines transformers with few-shot learning, detects objects with limited training data, end-to-end framework	44.9% AP [0.5:0.95] on MS COCO [101]
Person tracking	Deep Simple Online and Real-time Tracking (DeepSORT)	Combines deep appearance features, Kalman filtering for motion prediction, robust to occlusions but slower due to deep feature extraction	MOTA: 61.4% on MOT16 [102]
	FairMOT	Unifies detection and ReID into a single network. Balances detection and tracking accuracy	MOTA: 68.7% on MOT16 [102]
	ByteTrack	Simple, efficient, and robust in crowded scenes but relies heavily on detector quality	MOTA: 67.0% on MOT20 [102]
Person identification	Modified Centroid Triplet Loss (MCTL)	Transformer-based architecture with dual-branch design for multi-grained feature extraction, uses contrastive learning for unsupervised Re-ID	mAP: 98.6% on Market1501 [103]
	Transformer-based Multi-Grained Feature (TMGF)	Advanced loss function based on centroid triplet loss, emphasizes inter-class separation and intra-class compactness	mAP: 91.9% on Market1501 [103]
Face recognition	DeepFace	High accuracy, deep learning-based, uses 3D face alignment, large-scale facial dataset training	74% Accuracy [104]
	FaceNet	Employs triplet loss, maps faces to Euclidean space, efficient for verification, recognition or clustering	97% Accuracy [104]
	ArcFace	Angular margin loss, state-of-the-art accuracy, robust to variations in pose/illumination.	98% Accuracy [104]
	VGGFace	Pre-trained on large datasets, strong feature extraction capabilities	78% Accuracy [104]

Note: mAP denotes mean average precision, which calculates the mean of AP values across all classes or queries, measuring how well a model ranks relevant instances in retrieval tasks.

Modern CNNs like YOLO [105] have become popular for passenger analysis monitoring. Ref. [106] demonstrates the use of the YOLOv8 neural network to monitor passenger conditions, such as drowsiness, unfastened seat belts, and driver distractions, including emotion recognition, noticing its wide range of applications. Another study [107] explores the use of YOLOv3 for monitoring seat availability on buses, helping control transport occupancy statistics. In ref. [108], the authors showed the possibility of using multi-task learning methodology on the YOLOv8 architecture to enhance the classification of faces and their attributes. Another research direction has explored combining YOLO-based detection with advanced sensors, including thermal cameras, fisheye, depth, and other cameras. Ref. [109] showed that YOLO-fastest-xl combined with a fisheye camera achieves remarkable accuracy in detecting passengers inside vehicles. Consequently, these studies emphasize the effectiveness of various YOLO neural network versions in real-time monitoring.

Another notable deep neural network architecture is Faster R-CNN [110], which has shown promising results in human detection across various scenarios. As a region-based network built upon a CNN architecture that leverages a two-stage detection, Faster R-CNN excels in identifying objects within images. For instance, in the study by [111], the authors utilized Faster R-CNN to detect human body parts, enhancing human pose estimation and improving human–robot interaction. Similarly, another study [112] demonstrated the outperforming performance of this network when using a multi-branch architecture for pose estimation. This methodology can also be adapted for human detection. For example, in the work presented in [113], the authors used Faster R-CNN with different types of ResNet feature extractors to effectively detect people outdoors and indoors. Additionally, several papers have explored modifications to the Faster R-CNN architecture to enhance its performance in this domain. One such example is the paper by [114], in which the author proposes a self-enhanced approach for semi-supervised human detection, enabling training on labeled and unlabeled data.

Also, Single Shot MultiBox Detector (SSD) comes up as an efficient and unified framework for object detection that uses a single deep neural network to predict object categories and bounding box adjustments directly from feature maps at multiple scales [115]. Unlike methods relying on object proposals, SSD eliminates the need for resampling stages, making it faster and simpler to train while maintaining high accuracy. With its ability to handle objects of various sizes through multi-scale feature maps, SSD achieves state-of-the-art performance on benchmarks.

**Figure 4:**
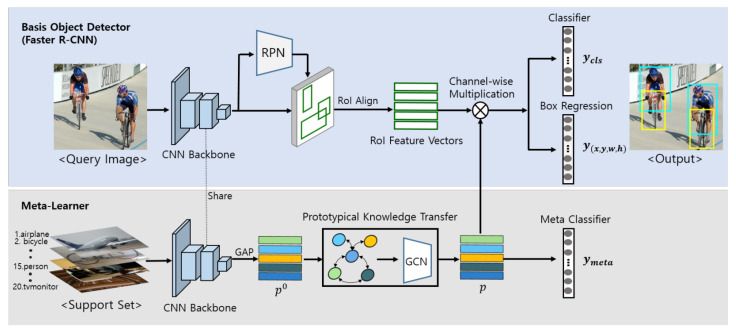
The architecture of the FSOD-KT network [117].

Another example is applying few-shot learning in the transformer-based model FS-DETR, developed by the authors in [116]. This model utilizes a few-shot learning algorithm to detect new objects without the need for fine-tuning, effectively outperforming previous models in the task. The innovative approach of FS-DETR demonstrates the potential of combining transformer architectures with few-shot learning techniques. Few-shot learning approaches leverage meta-learning, where models are trained across various tasks to develop strategies for quickly adapting to new tasks. For instance, in ref. [117], the authors present a neural network architecture designed explicitly for object detection. This architecture utilizes a meta-learner to generate prototypes, which the model then employs to classify and make a regression of objects based on the R-CNN framework (see Figure 4).

Many studies use the Deep Simple Online and Real-time Tracking (DeepSORT) [118] algorithm to track people. This algorithm works well in real-world conditions and accurately predicts the next position of tracked objects. DeepSORT combines deep learning-based object detection models such as YOLO with a re-identification algorithm to match predicted objects with previously tracked ones. When integrated with YOLO, this approach demonstrates high performance in both accuracy [119] and computational efficiency [120,121]. Moreover, DeepSORT has proven effective in crowd-tracking applications where it is necessary to predict the movement trajectories of a large number of people within a single video frame [122].

For re-identification tasks, we can underline the Modified Centroid Triplet Loss (MCTL) method, which improves how models learn to tell different people apart [123]. Instead of just comparing single images, MCTL focuses on making the features of the same person more similar while pushing the features of different people further apart. It does this by pulling features closer to their class center and pushing them away from other class centers, helping the model learn more distinct and accurate features for better performance. Another method is the Transformer-based Multi-Grained Feature (TMGF) approach, which focuses on extracting detailed features from images for unsupervised Re-ID [124]. It uses a modified Vision Transformer (ViT) as its backbone and adds a dual-branch architecture to capture global and local (part-level) features. The global features represent a person’s overall appearance, while the part-level features focus on more minor details like clothing patterns or accessories. These features are learned using contrastive learning techniques, which help the model distinguish between different individuals without needing labeled data. During testing, only the global features are used for matching, making the process efficient while still achieving strong performance. These two methods are on the top of supervised and unsupervised methods in ReID algorithms now, showing only a 6% difference [103].

Many deep learning models need large datasets to achieve high accuracy. However, such datasets are not always publicly available, leaving researchers to work with limited data. To address this challenge, few-shot learning methods used to enable AI models to generalize effectively, even when trained on smaller datasets [125]. These approaches facilitate the training of models by allowing them to learn from new data while retaining previously acquired knowledge, thereby enhancing their performance in data-limited environments. A notable example is described in ref. [126], where the authors develop a framework for human tracking using a Siamese network. This innovative approach involves suggesting potential human candidates and then employing a few-shot learning algorithm to classify the IDs of the detected individuals.

One of the first deep learning models for face recognition, DeepFace used a nine-layer neural network and achieved human-level accuracy on face recognition tasks, marking a significant milestone in the field [127]. Then, Google showed that FaceNet utilized a deep convolutional neural network (DCNN) with a triplet loss function to map face images into a compact embedding space, where Euclidean distances directly reflected face similarity, setting a new standard for face recognition [38]. Another model had a VGGNet architecture; VGG-Face demonstrated the effectiveness of very deep convolutional networks for face recognition, achieving strong performance and highlighting the importance of depth in neural networks [63]. Similarly, ArcFace introduced an angular margin loss function to enhance the discriminative power of face embeddings, significantly improving performance on challenging face recognition benchmarks and surpassing previous state-of-the-art methods [59]. Comparing these methods, DeepFace demonstrated early success in deep learning-based face recognition; however, it lacked efficiency for large-scale applications. On the other hand, FaceNet set a new standard with its embedding-based approach, offering high accuracy and compact representations, but it required extensive training data. VGG-Face leveraged deep architectures for improved performance; nevertheless, it was computationally expensive. Therefore, ArcFace also had a disadvantage, which represented the cost of increased training complexity.

### 4.4. Unified Methods

Unified tracking and detection methods integrate object tracking and detection directions in a single structure, providing a more accurate and efficient processing algorithm in dynamic domains. Both tasks allow the user to create real-time applications where persistent object identification is essential.

One of the frameworks in this area is the method proposed in [128] using three types of ResNet-based neural networks for each task: detection, tracking, and recognition to create an efficient structure. In addition, a hierarchical Gaussian process was used to develop such a framework for human detection and tracking, which can improve accuracy by incorporating prior knowledge for combined tasks [128]. The combined method of the active testing (AT) paradigm with Bayesian filtering shows a robust algorithm for detection and tracking in conditions of the loss of an object on the camera for a short time [129].

As a result, based on analysis, we can see that different CV tasks require specialized models designed to address the unique challenges and objectives of each problem. Certain models are better at extracting specific features, managing scenes with many people, and recognizing people in various scenarios. Some models can be used uniformly or in combination with other CNNs, but some models can be used only for specific tasks. For example, it is possible to build a detection, tracking, and recognition unified system like in [128] if models have mutual compatibility between input and output data. However, another challenge, like computational efficiency, can appear for such hybrid systems [130]. Depending on the requirements of each task, the architectures and training approaches differ significantly, utilizing various network topologies, loss functions, and processing strategies to achieve the best possible performance.

### 4.5. Evaluation Metrics

When developing detection, tracking, and recognition systems, selecting the appropriate evaluation metrics is essential for accurately assessing model performance and comparing it to state-of-the-art benchmarks. This section outlines some of the most commonly used metrics for these tasks.

For people detection, which is similar to object detection, the most widely used evaluation metric is Mean Average Precision (*mAP*). This metric calculates the average precision across different Intersection over Union (IoU) thresholds, providing a robust measure of model accuracy:

$$mAP=\frac{1}{N}\sum_{i=1}^{N}AP_{i}$$

where *N* represents the number of classes. In our case, since the model is designed for human detection only, *N* is equal to 1. Examples of studies utilizing these metrics include [95,96,99].

Evaluating the effectiveness of people tracking has previously focused on the basic metrics of accuracy and precision [131]. With the development of this field, new metrics have emerged. One of them is the multiple objects tracking accuracy (*MOTA*) metric [131], which offers an overall indicator of how successfully a tracker can continue to provide precise and reliable tracking. It accounts for errors such as false negatives (*FNs*), false positives (*FPs*), and identity switches (*IDSs*). The *MOTA* formula is as follows:

$$MOTA=1-\frac{\sum_{t=1}^{N}FN_{t}+FP_{t}+IDS_{t}}{\sum_{t=1}^{N}GT_{t}}$$

where *GT* represents the number of ground truth objects. *MOTA* is valuable for comparing different models in terms of tracking reliability. Another key metric is *IDF*1, which addresses some of the limitations of *MOTA* by focusing on how well the model maintains correct identity tracking over time. It is calculated as the ratio between true positive identities (*IDTP*), false positive identities (*IDFP*), and false negative identities (*IDFN*):

$$IDF1=\frac{2*IDTP}{2*IDTP+IDFP+IDFN}$$

For a comprehensive evaluation, researchers suggest using multiple uncorrelated metrics, combining measures of accuracy and robustness to capture all sides of this complex task [132]. For face verification, common evaluation metrics include precision, accuracy, Equal Error Rate (EER), and *F*1-score. The EER measures the rate at which false acceptance and false rejection rates are equal, making it particularly useful for video-based systems. The *F*1 score, which balances *precision* and *recall*, is given by the formula:

$$F1=\frac{2*Precision*Recall}{Precision+Recall}$$

In summary, the choice of evaluation metric depends on the specific task and dataset. A diverse set of metrics ensures a more reliable assessment of model performance.

## 5. Applications

### 5.1. Crowd Counting

**Figure 5:**
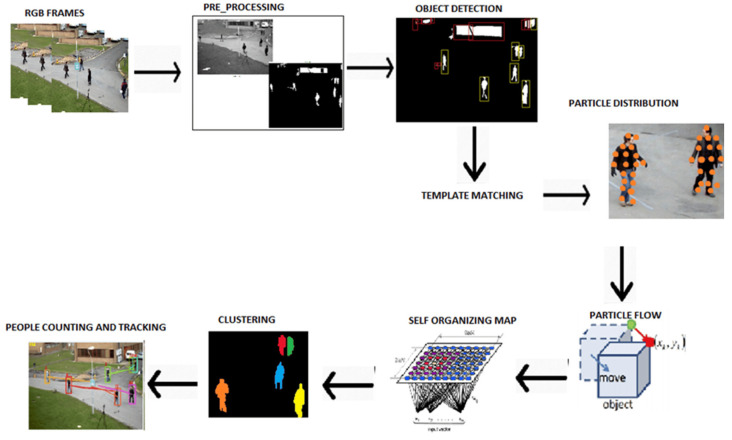
The architecture of the proposed people counting and tracking system [79].

Crowd counting is an important task in computer vision, with numerous applications in public safety, event management, urban planning, and resource allocation. It entails precisely estimating the number of individuals in an image, which is frequently captured in complex and densely populated scenes. The proposed work [79] creates a precise, long-lasting, and effective system that can count and track people with minimum errors in public spaces. The whole system consists of preprocessing stages, an object detection part, people verification, PFs (particle flows) and feature extraction, SOM (self-organizing map)-based clustering, then people counting and people tracking stages (see Figure 5).

In the proposed system, area-based filtering is used for object detection, and multiple filters are applied to smooth out images and eliminate noise. The next step is discretion of the remaining objects, and those whose sizes fall within the given range are regarded as detected. Following object detection, five to six templates are selected from each dataset, and the objects are confirmed to be people using template matching. Additionally, moving objects’ particle flows (PFs) are extracted, and their features are combined with a modified self-organizing map (SOM). The number of PF clusters and the number of people in an image correlate, which is the basis for people counting. Following the detection of humans, the trajectories are created and monitored appropriately. In ref. [133], researchers proposed an IoT system with surveillance cameras to gather video data for a pre-trained model. In their architecture, the cameras should be in designated public spaces, giving each one an IP address. After that, the cameras will be linked to the cloud via Wi-Fi technology. The next step involves connecting the user’s device to the cloud. The data are then sent from the cloud using the DCNN training model to primarily run the crowd counting system after accessing the data from the cloud to the end users’ devices via the internet. The necessary processed data will then be gathered. Lastly, the outcome will be transmitted and shown on the user’s mobile app.

### 5.2. Security

**Figure 6:**
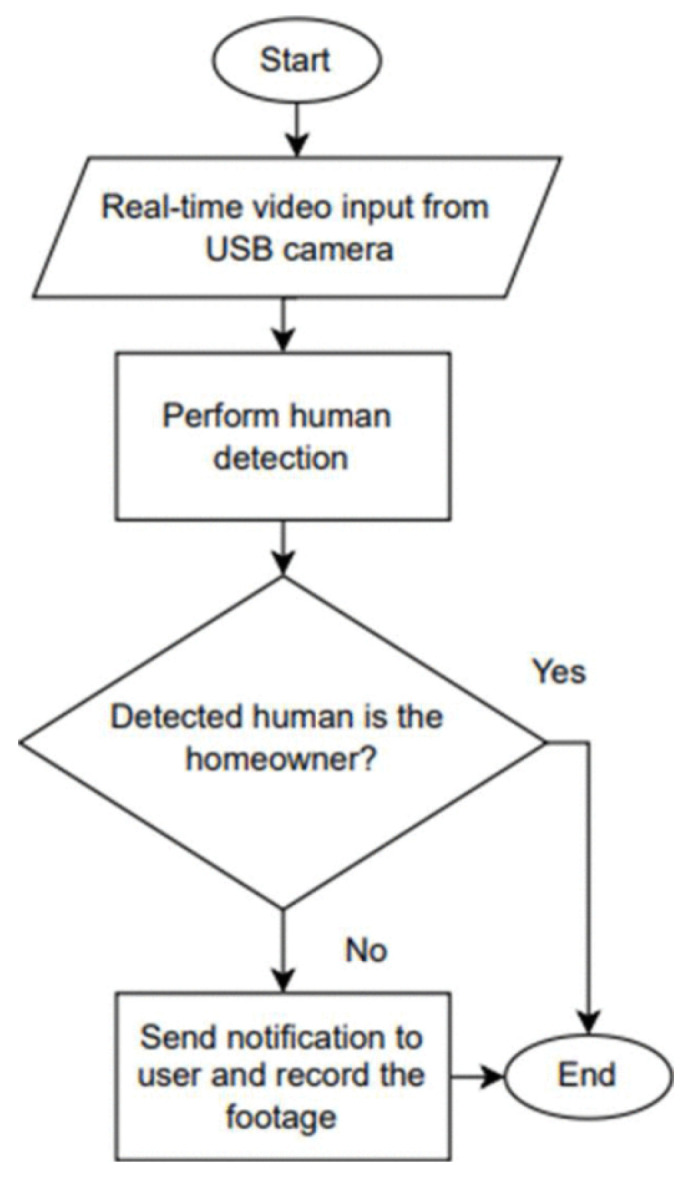
Flow chart of the intruder detection system [134].

Modern privacy and security systems rely heavily on person detection and identification technologies, which use person detection, identification, and tracking technological breakthroughs to improve monitoring, access control, and surveillance. One study shows how deep learning in surveillance can detect home intruders and homeowners [134]. Authors used the MobileNets model, which helps to build mobile and embedded vision applications for figure recognition. Also, EfficientDet is used for object detection tasks to categorize a person as either a homeowner or an unidentified individual. Raspberry Pi 4 (Pi) helps this system perform video surveillance and detection with classification in real-time. The homeowner will receive a notification if an intruder is found, along with a brief video recording of the incident that can be viewed through a web application (see Figure 6).

**Figure 7:**
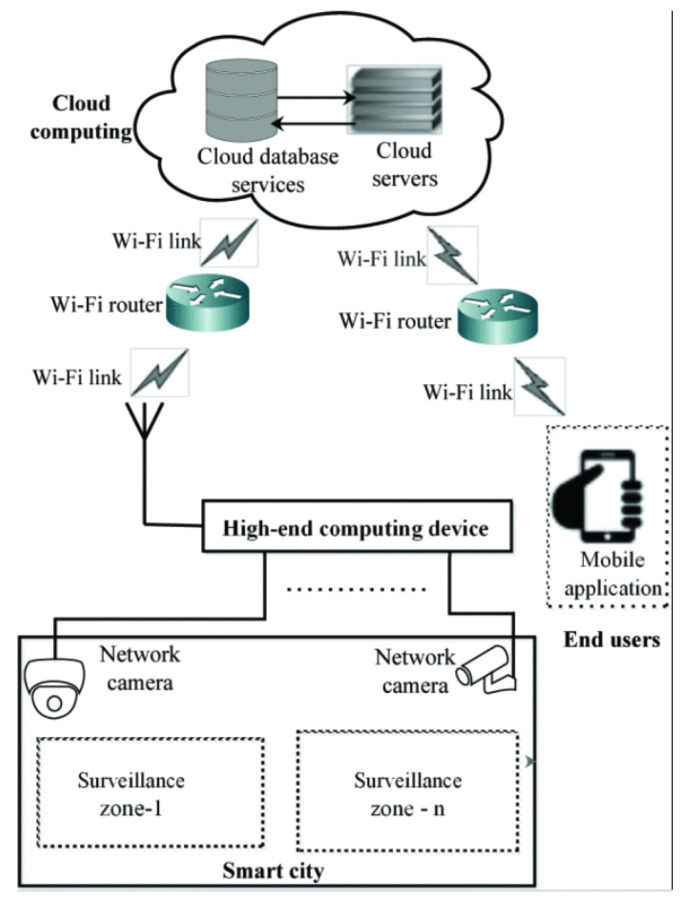
The proposed model for the loitering detection system [135].

Another application is in the proposed Loitering Detection System (LDS) with re-identification (ReID) capability, which can function across several camera feeds in real-time [135]. It tracks people using DeepSORT and detects them using the YOLOv3 algorithm. In this crowd counting system, live video streams are captured by high-resolution network cameras (Hikvision 4 MP). Videos are processed on a high-end computing device like Dell Tower Workstation, equipped with the necessary experimental setup. For wider accessibility, the processed data are sent to cloud services via a platform such as Twilio, which offers communication services with a limited number of free credits for research. Lastly, the user uses a Python-based interface that provides both offline and online video processing modes to interact with the system. Research showed that an interface allows users to select the surveillance zone and set time and displacement thresholds, either by choosing predefined values or customizing them for specific applications (see Figure 7).

Cross-camera re-identification, however, presents several difficulties. These include occlusions that could mask important identifying characteristics and changes in lighting between camera feeds, which can alter how people appear. Furthermore, it may be challenging to reliably match people across several feeds due to camera angles, resolutions, and background variations.

With the increasing deployment of face recognition systems in public and private spaces, privacy concerns are growing. Traditional face recognition systems rely on centralized databases where facial embeddings are stored, making them vulnerable to data breaches. Research into privacy-preserving face recognition using techniques like federated learning and homomorphic encryption is essential for protecting user data while maintaining system utility [136]. They also require large datasets for training, including datasets with larger angle face picture samples gathered [137], but in many real-world applications, acquiring vast amounts of labeled face data is impractical. To address this, one-shot and few-shot learning methods, such as Siamese networks and matching networks, have gained popularity. These approaches aim to recognize individuals based on a single or very few examples [138], making them more applicable in surveillance and biometric identification.

Despite significant advancements in person detection and identification technologies, research gaps remain in balancing accuracy with computational efficiency, ensuring reliable performance in challenging environments.

### 5.3. Smart Cities and Transport

Person detection and identification technologies are essential components of smart city and transportation systems, improving traffic management and public safety. Authorities can track people across multiple cameras in public transportation using techniques like re-identification, which enhances their capacity to spot suspicious activity and improve passenger management in transport. Ref. [139] presents an advanced architecture for a camera-based monitoring system designed for smart city roads, specifically utilizing people detection and tracking. The core pipeline begins with Nvidia DeepStream software, enabling the deployment of a real-time detection model across city-installed cameras. This model identifies people and objects, such as vehicles, and then generates multiple data streams for parallel processing. In this setup, video streams for people detection operate alongside heatmaps and density analysis to provide insights into crowd distribution and vehicle presence. Kafka is implemented as a message broker to minimize the latency between real-time outputs and analytics processing, ensuring that data flow seamlessly to connected applications. Grafana then retrieves these data from the Kafka server, using them to create a live, interactive dashboard that visualizes analytics generated by the system, such as density metrics for pedestrians and vehicles. This integrated architecture enables real-time monitoring, making it highly suitable for traffic and pedestrian management in urban areas.

Almaty, Kazakhstan’s capital, has launched a facial recognition payment pilot in its subway system in collaboration with one of the second level bank and its Face Pay software [140]. Initially, passengers can use the service at two stations, with plans to expand to more stations over time. The system ensures 100% security, with passenger information stored by the bank and no personal data being accessible to metro or bank employees. The Almaty metro serves over 100,000 passengers daily. This initiative is part of Kazakhstan’s broader investment in biometric technologies aimed at advancing digital government services in the region.

To design a system with low computational costs for monitoring helmet compliance among motorcycle riders, the authors of ref. [141] show an efficient architecture. This system centralizes the data collection from various cameras onto a cloud server, enabling the integration of multiple video streams into a single detection framework. In the proposed architecture, all training processes are conducted on a powerful GPU-enabled centralized server, which significantly reduces the computational cost on individual client devices. This setup allows the system to manage and analyze data from multiple cameras simultaneously without requiring high processing power at each camera location. Communication between client devices and the server is established using JSON over HTTP, allowing for efficient data exchange and integration. This design allows for seamless processing of video streams, allowing for real-time monitoring and verification of helmet compliance for motorcycle riders on the road.

Real-time face recognition in large-scale systems, such as those used in airports or smart cities, requires efficient and scalable solutions. Current state-of-the-art models can be computationally expensive, limiting their deployment in resource-constrained environments. Model compression techniques (e.g., quantization, pruning) and hardware accelerators (e.g., GPUs, TPUs) are being explored to reduce latency and increase throughput [59].

Smart cities and transportation areas have similar problems in terms of balancing accuracy with computational efficiency. This is especially true when the hardware is limited or data streams are extensive.

## 6. Results and Discussion

From the review above, we can conclude that the classical methods are still in use in these studies, but their performance significantly drops in complex environments with varying lighting, occlusions, and high traffic, highlighting their limitations in large-scale, real-world applications. Classical methods such as HOG-SVM and optical flow are computationally less intensive compared to deep learning approaches, making them suitable for low-resource environments. The development of CNN architectures gave a boost for the development of systems for person detection, tracking, and verification. However, for real-time processing, CNN networks should be used in combination with RNN layers to extract temporal information. A promising approach for crowd analysis involves the integration of crowd-specific models like CSRNet, which leverages density maps to effectively estimate crowd sizes and distributions [142]. Furthermore, the growing adoption of vision transformers in computer vision tasks presents another avenue for innovation, as these models can be adapted to enhance performance in this domain.

CNN-based architectures like YOLO and its various versions have set new standards for accuracy and speed, especially in real-time applications, making them ideal for high-traffic environments like public transportation and surveillance systems. Application of CNN methods in security showed significant advancements in person detection and identification technologies. In addition to this, Unified methods also showed promising results. However, deep learning models are often computationally intensive and require lightweight architecture and hardware optimization. Therefore, there are still research gaps in areas like striking a balance between computational efficiency and accuracy to guarantee dependable performance in demanding environments. Advanced systems and architectures proposed in smart cities and transportation areas, including real implementation in public transport, might have difficulty with scaling and adjusting to quickly changing environments, such as congested public areas, erratic weather patterns, or various lighting situations. Some research has shown that combining deep learning models with multimodal sensors (such as thermal cameras, fisheye cameras, and depth cameras) improves detection accuracy, especially in challenging environments such as poor lighting or areas with high population density. Unified models that combine detection and tracking improve efficiency in multi-task scenarios. However, the complexity of integrating the multitask approach into unified approaches can lead to decreased accuracy in individual tasks. The social impact of technologies that involve detecting and monitoring of people’s movements must include broad social aspects and trade-offs between accessibility and comfort, security and privacy [143]. Such technologies are primarily aimed at improving safety in public places where people gather, but such technologies also raise some concerns about the vulnerability and insecurity of people [144]. All these factors influence the acceptance of these technologies in society, so these social aspects need to be taken into account when developing and designing these systems. It is also important to inform all monitoring participants about possible risks and benefits. Therefore, the topic of ethical considerations is important when using new technologies, but most articles do not cover this aspect in detail. Technological advances, especially in artificial intelligence, computer vision and data science, raise a number of ethical concerns, from data privacy to algorithmic bias and impact on society. Studying the impact of these technologies on each ethical aspect can help in the future to create more reliable and trustworthy technologies for implementation in real-world settings. Incorporating ethical considerations into technology development not only reduces potential risks but also promotes responsible practices that prioritize human dignity, justice, and the public good.

In terms of datasets, despite the availability of well-established data sources, we see that some widely used datasets have been discontinued, reducing resources for comprehensive, privacy-preserving research. The PeopleSansPeople generator provides a powerful tool to minimize data privacy risk, as it produces synthesis data. However, models trained solely on synthetic data often require further fine-tuning on real-world datasets such as COCO to handle natural environmental variation. Thus, future synthetic datasets can improve their realism by better simulating natural conditions. A critical aspect of datasets is ensuring diversity and representativeness to minimize bias and enhance generalization across different demographics and environments. To address the environmental aspect, synthetic datasets can be improved by incorporating the ability to generate diverse environmental factors, such as varying lighting conditions, weather patterns, and occlusion scenarios, to better align with real-world challenges. While many analyzed datasets aim to include balanced representation across genders and age groups, further enhancements in generalization can be achieved through collaboration with experts in demographic analysis. Such interdisciplinary efforts can help create more inclusive datasets, ultimately improving the performance and fairness of models across diverse populations.

## 7. Challenges and Future Trends

As methods for person detection, tracking, identification, and face recognition continue to improve, several challenges remain that will shape the direction of future research. One of these issues is real-time application. Despite the fact that YOLO and CNNs have shown to be successful, it is still difficult to maintain high accuracy while satisfying real-time requirements such as minimum computational resource consumption and fast and accurate detections, particularly in complicated situations with numerous moving subjects. This is an important topic for future study since real-time activities demand not only fast and accurate models but also optimization strategies to minimize detection and classification latency.

Another challenge is the synchronization of multi-domain and multiple-camera systems, especially in the complex scenario of integrating and synchronizing these cameras in real-time, which presents major algorithmic and computational challenges. On the other hand, achieving consistent and accurate detection across complex camera angles and modalities is important, especially for applications where occlusion and varying lighting conditions can impact accuracy. As we discussed before, some articles use thermal cameras with DL models, but the possibility of extending applications with fisheye, depth, and other cameras still is the major challenge. Addressing these issues will be key to improving system performance and reliability.

To improve the robustness and efficiency of real-time human detection, tracking and identification systems, future research will build on the foundation discussed in this review. Future work should focus on optimizing deep learning models for real-time tasks, improving multi-camera synchronization, and creating a new algorithm of a system that can better handle the complexities of real-world environments.

## 8. Conclusions

This study highlights the tremendous advancements in face recognition, tracking, identification, and person detection technologies, especially with the use of CNNs and other deep learning models. Even though these systems are now better able to handle common problems like variations in lighting, partial occlusion, and appearance variations, our review identifies important research gaps that still require attention. More specifically, these systems’ accuracy and reliability are still limited by persistent occlusions and the challenge of telling visually similar people apart. For the YOLO state-of-art model itself, minimal computational resource consumption and fast, accurate detections remain difficult, particularly in complex scenarios with multiple moving people. Also, we pointed out the significance for future studies incorporating multi-modal data to enhance robustness in dynamic environments, like thermal or depth imagery. In order to close these research gaps and ensure the continued success of person tracking and identification technologies, more sophisticated algorithms and extensive testing under a range of real-world scenarios are required.

## Figures and Tables

**Table 1:** Statistics of the popular datasets.

Task	Dataset	Images	Image Format and Example	Labels	Performance Metrics
Human detection	AI City Challenge (AIC) dataset for motorbike helmet violation detection [44]	20,000 frames	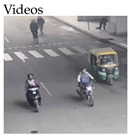	BB	mAP@50: 48.6 [44]
	PeopleSansPeople [45]	500,000	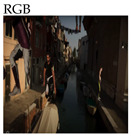	BB with keypoints, semantic segmentation	mAP@50: 86.2 [45]
	COCO [46]	200,000	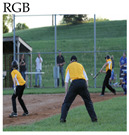	BB with keypoints, segmentation map	mAP@50: 65.9 [47]
Human tracking	MOTChallenge [48]	17,757 frames	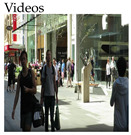	No	MOTA = 80.7, ID F1 score = 82.2 [49]
	SportsMOT [50]	150,000 frames	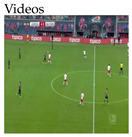	BB	MOTA = 97.1 [51]
	PoseTrack [52]	66,374 frames	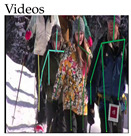	15 body keypoints with id	MOTA = 64.09 [53]
Human segmentation	Cityscapes [54]	25,000	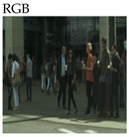	Pixel-wise annotations and coarse annotations	mask AP = 38.0 [55]
	COCO [46]	200,000	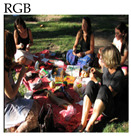	Pixel-wise annotations	mask AP = 56.1 [56]
	Segment Anything 1 Billion (SA-1B) [57]	over 1 billion images	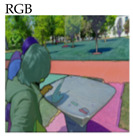	Mask-based annotations	mask AP = 42.8 [57]
Face recognition	Labeled Faces in the Wild (LFW) [58]	13,233	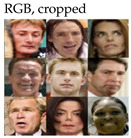	BB with identity	Accuracy = 99.83% [59]
	CelebA [60]	200,000	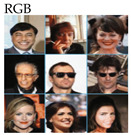	BB with identity	Accuracy = 82% [61]
	YouTube Faces DB [62]	3425 videos	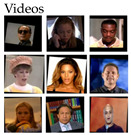	BB with identity	Accuracy = 98.02% [59]
	VGGFace [63]	2.6 million	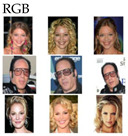	Cropped images with identity	Accuracy = 98% [64]

Note: BB denotes the bounding box, which represents the four coordinates of the object region.

**Table 2:** Classical computer vision-based methods mapping to primary problem domains.

Domain	Method	Key Features	Performance Metrics
Person detection	Histogram of Oriented Gradients (HOG)	Edge-based, robust to lighting, sliding window, high-dimensional, used with SVM	AP: 0.16 on PASCAL VOC [71]
	Deformable Part-Based Model (DPM)	Part-based, handles pose and occlusion, uses HOG, hierarchical, computationally heavy	AP: 0.34 on PASCAL VOC [71]
Person tracking	SDOF-Tracker (based on optical flow)	Motion-based, frame-to-frame tracking, sensitive to lighting and noise	MOTA: 46.7% on MOT20 [72]
	Kalman filter	Predictive, good for smooth motion, needs external detection	MOTA: 35.4% on MOT20 [73]
	Continuously Adaptive Mean Shift (CAMshift)	Color-based, efficient, adapt to scale, handle rotation, struggles with heavy occlusion	MOTA: 59.2% on urban road intersection and highway monitoring video [74]
Person identification	Ensemble RankSVM	Ranking-based, feature-dependent	Rank-1: 14% on VIPER [75]
	Symmetry-Driven Accumulation of Local Features (SDALF)	Exploits symmetry in the human body for feature extraction and matching	Rank-1: 20% on VIPER [76]
	Custom Pictorial Structures (CPS)	Pose-based, fails with occlusion	Rank-1: 21.8% on VIPER [77]
Face recognition	Eigenfaces	PCA-based, holistic, sensitive to lighting and pose	96% Accuracy on SCD 2500 [78]
	Fisherfaces	LDA-based, discriminative, better with lighting variation	94.12% Accuracy on ORL [78]
	Gabor wavelets	Texture-based, robust to lighting and expression	67.6% Accuracy on FERET [78]

Note 1: AP denotes the average precision metric, where higher AP scores indicate better detection performance. Note 2: MOTA denotes multiple object tracking accuracy. Higher MOTA scores indicate better tracking performance, which means fewer errors in tracking objects across frames. Note 3: Rank-1 denotes the percentage of queries where the correct match for a person is found as the top result in the ranked list of candidates. Note 4: SCD stands for self-created dataset with number of images.

## Data Availability

The original contributions presented in this study are included in the article; further inquiries can be directed to the corresponding authors.
